# Diversity, phylogeny, and bathymetric zonation of *Sirsoe* (Annelida: Hesionidae) from colonization experiments in the South China Sea, with the description of three new species

**DOI:** 10.1002/ece3.10256

**Published:** 2023-07-17

**Authors:** Yadong Zhou, Yuru Han, Wei Xie, Mingting Li, Zhi Wang, Dongsheng Zhang

**Affiliations:** ^1^ Key Laboratory of Marine Ecosystem Dynamics Second Institute of Oceanography, Ministry of Natural Resources Hangzhou China; ^2^ Southern Marine Science and Engineering Guangdong Laboratory (Zhuhai) Zhuhai China; ^3^ School of Marine Sciences Sun Yat‐sen University Zhuhai China; ^4^ State Key Laboratory of Marine Environmental Science, College of Ocean and Earth Sciences Xiamen University Xiamen China; ^5^ School of Oceanography Shanghai Jiao Tong University Shanghai China

**Keywords:** biogeography, deep sea, metabarcoding, organic fall

## Abstract

The South China Sea (SCS) basin is hypothesized to host distinct and bathymetrically differentiated fauna due to its semi‐enclosed basin and three‐layer circulation system. To test this hypothesis, three cow falls are artificially deployed at separate depths (655, 1604, and 3402 m) on Zhongnan seamount in the middle SCS, and the associated worms, *Sirsoe* spp. are selected as targets to explore their diversity, phylogeny, and zonation pattern. Analyses of collected specimens reveal three new *Sirsoe* species, which were then nominally described and named as *S. polita* sp. nov. (655 m), *S. nanhaiensis* sp. nov. (1604 and 3402 m), and *S. feitiana* sp. nov. (3402 m), and one known species (*S. balaenophila* lineage II). Metabarcoding analyses on cow‐fall sediments reveal seven additional Operated Taxonomic Units (OTUs) assigned to *Sirsoe*, increasing the *Sirsoe* diversity to 10 species/OTUs in the middle SCS. Their distribution along depth shows increasing diversity toward the deeper sites. Phylogenetic inferences recover *S. polita* closely related to *S. alucia* from the Southwest Atlantic, forming a lineage deeply divergent from others. The nine deep‐water species/OTUs are scattered in three distinct lineages showing closer phylogenetic relationships between 1604‐ and 3402‐m counterparts. The lineage formed by *S. naihaiensis* and *S. feitiana* is distinct from other non‐SCS congeners both morphologically and genetically. These results suggest multiple independent invasions of *Sirsoe* to the SCS, a new lineage potentially endemic to the SCS, and a strong zonation pattern related to depth, especially between the shallow (655 m) and the deep (1604 and 3402 m) sites. The semi‐enclosed feature combined with the physical structure of the SCS may contribute to such a pattern. This work is registered in ZooBank under: urn:lsid:zoobank.org:pub:317771C8‐42D717‐4765‐A168‐B3BE99B09FBF.

## Graphical abstract

A combination of specimen examination, metabarcoding, and phylogeny was used to explore the diversity of *Sirsoe* from colonization experiments in the South China Sea. A total of 10 species/OTUs were recovered, and their phylogenetic positions were resolved, with three new species described as well. A zonation pattern was observed.

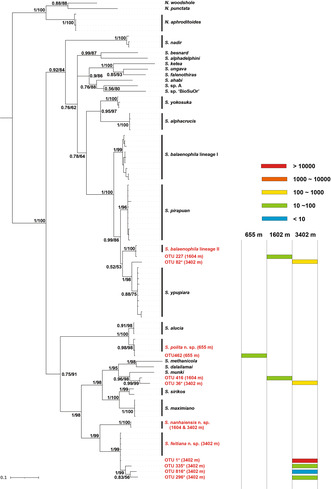

## 1 INTRODUCTION

As the largest marginal sea in the tropics, the South China Sea (SCS) has a semi‐enclosed basin bordered by the Asian continent to the north and west, and separated from the West Pacific by Taiwan, Philippine Islands, and the Greater Sunda Islands. Water exchanges between the SCS and its surrounds are mainly through the Luzon Strait (LS), where a vertical sandwich‐like inflow–outflow–inflow is formed in the upper–middle–deep layers. Water transport through LS is much stronger in the upper layer than in the middle layer. The deep layer lacks direct water exchange due to an isolated basin of deep SCS which is prevented by the sill in the LS (~2400 m) (Cai et al., 2020; Gan et al., 2022). In the context of its geologically semi‐enclosed nature and strongly stratified physical structure, we hypothesize that the deep SCS harbors benthic invertebrate with a high level of endemism and depth zonation pattern, as what is observed in the Mediterranean Sea (Danovaro et al., 2010), and lineages deeply divergent from relatives outside. And a taxa group with members colonizing different depths can be used to test these hypotheses.

So far, little is known about the deep‐sea biodiversity in the SCS, except for cold‐seep fauna and cold‐water corals from a few sites (Dong et al., 2021; Li & Wang, 2019; Zhao et al., 2020). However, they cannot be used to test these hypotheses due to a limited depth range of the locations and/or a lack of genetic data. Recently, a small cetacean fall has been reported from a SCS seamount (Xie et al., 2023), evidencing the existence of such kind of habitats in the region for the first time despite their worldwide distribution (Smith et al., 2015). Investigation of cetacean falls usually recovers a wide variety of animals which can hardly be encountered when surveying the background environments (such as ocean basins and seamounts) and shows strong reliance on such habitats rich in nutrients and energy, thus revealing “hidden” biodiversity in a region (Smith et al., 2015). Although natural cetacean falls can only be encountered by chance, artificially implanted vertebrate carcasses, mimicking whale falls, at different depths provide alternative methods to study fauna associated with organic falls and an opportunity to test the hypothesis on the SCS biogeography (Amon et al., 2014, 2017; Braby et al., 2007; Fujiwara et al., 2007).

Animals of different nutrition modes emerge at cetacean falls in a successional sequence of stages: mobile‐scavenger stage, enrichment‐opportunist stage, sulfophilic stage, and reef stage (Smith et al., 2015). Among them, *Sirsoe* and *Vrijenhoekia* from the family Hesionidae are two important opportunists recovered from a wide variety of depth ranges (Pleijel et al., 2008; Shimabukuro et al., 2019). The two genera show high similarity to each other in both morphology and genetics, and were initially recovered as sister clades in early phylogeny (Rouse, Carvajal, et al., 2018; Summers et al., 2015). The main morphological characters distinguishing them were attributed to the absence of a median antenna, the presence of glandular lip pads (GLP), and well‐developed neuropodia starting on segment 4 in *Vrijenhoekia* rather than *Sirsoe* (Pleijel et al., 2008). However, this diagnosis was challenged by subsequent discoveries of *Vrijenhoekia* members with median antenna and paraphyletic status of those diagnostic features in a phylogeny with wider taxa sampling (Shimabukuro et al., 2019). Thus, Shimabukuro et al. (2019) adapted the diagnosis of *Sirsoe* to accommodate members in *Vrijenhoekia* and synonymized the two genera, resulting in a monophyletic *Sirsoe*, which is accepted in the present study.

Currently, *Sirsoe* species are mainly reported from whale falls from the Northeast (NE) Pacific and the Southwest (SW) Atlantic close to the Brazilian coast (Pleijel et al., 2008; Rouse, Carvajal, et al., 2018; Shimabukuro et al., 2019; Summers et al., 2015), and scattered records are also from hydrothermal vents and cold seeps from the Gulf of Mexico, off Costa Rica coast, Caribbean Sea, Okinawa Trough, and Mariana Trough (Blake, 1991; Desbruyères & Toulmond, 1998; Pleijel, 1998; Rouse, Carvajal, et al., 2018; Rouse, Goffredi, et al., 2018). A phylogenetic study revealed three clades in this genus, all of which, however, did not cluster by neither zoogeographic zones nor habitat types, except for three seep species forming a subclade in clade I (Shimabukuro et al., 2019). Most species seemed to be locally distributed, while inter‐basin distribution was also confirmed in *S. balaenophila* “stricto sensu” and *S. siriko*, and depth might play an important role in diversification between *S. alphacrucis* and *S. yokosuka* (Shimabukuro et al., 2019). However, the western Pacific species were absent from available phylogenetic studies due to a lack in molecular data, leaving large gaps in knowledge of their biogeography.

Recently, we found abundant *Sirsoe* worms associated with implanted cow falls at three depths on Zhongnan seamount in the SCS. Here, we combine the specimen examination, phylogenetic and metabarcoding analyses to (1) describe and characterize the worms collected from cow falls; (2) evaluate species diversity of *Sirsoe* in the deep SCS; (3) examine if the SCS host lineages distinct from other regions and (4) test the role of depth in shaping their pattern in the SCS.

## 2 MATERIALS AND METHODS

### 2.1 Sample collection and preservation

**FIGURE 1:**
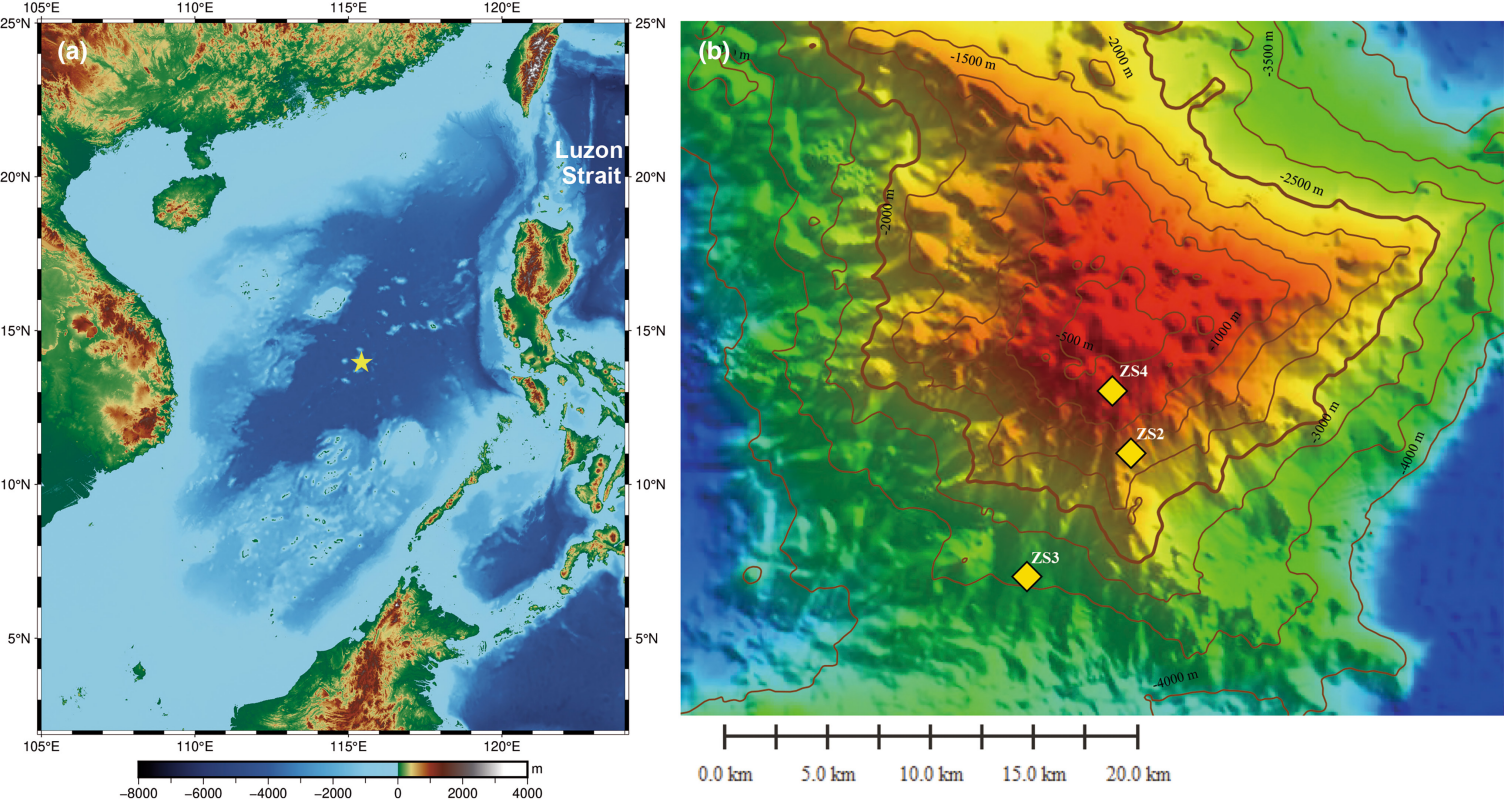
(a) Location of Zhongnan seamount in the South China Sea (indicated by yellow star); (b) Bathymetry of Zhongnan seamount with the three cow fall‐deployed sites marked by yellow diamond.

Zhongnan seamount is a conical seamount located in the middle SCS basin, with a depth range from 288 m at the top to 4355 m at the bottom (Figure 1). In March 2021, three cows (with all internal organs removed, about 800 kg on average) were deployed at separate depths on Zhongnan seamount in the SCS using a remotely operated deployer system during the TS2‐5 cruise of R/V *Tansuo2*.

We revisited the deployed cows with the manned submersible *Shenhaiyongshi*, during the TS2‐8 cruise of R/V *Tansuo2* in July 2021, about 4 months after the deployment. Dense aggregations of benthic animals were observed. Polychaete worms were collected using either a suction sampler or pushcore. Sampling information is detailed in Table 1. Most samples were fixed and preserved in ethanol, with a few frozen separately under −20°C.

**TABLE 1:** Information of deployment of cow falls and sample collection.

Stations	Latitude (°N)	Longitude (°E)	Depth (m)	Date of deployment	Date of sampling	No. of collected ind.
ZS2	13.915	115.430	1604	2021/3/19	2021/7/9	16
ZS3	13.861	115.385	3402	2021/3/20	2021/7/11	20
ZS4	13.942	115.423	655	2021/3/21	2021/7/13	59

### 2.2 Specimen repositories

Type specimens and material examined are deposited at the Repository of the Second Institute of Oceanography (RSIO), Ministry of Natural Resources, Hangzhou, China.

### 2.3 Morphology

Specimens are morphologically examined under a stereomicroscope (Zeiss Discovery V.16). Optical images are taken using a CCD camera mounted on the stereomicroscope. To show details of neurochaetae, scanning electron microscopic (SEM) images are obtained following methods described in Han et al. (2021).

### 2.4 Molecular and phylogeny

Tissues are dissected and used for DNA extraction with the E.Z.N.A. Tissue DNA Kit (Omega Bio‐tek) following the manufacturer's protocol. The universal primer pairs, HCO2198/LCO1490 (Folmer et al., 1994) and 16Sar/16Sbr (Palumbi, 1996) are used to amplify partial sequences of mitochondrial genes, c oxidase I (*COI*) and *16S* rRNA, respectively. Mixtures for Polymerase Chain Reaction (PCR) contained 1 μL of each primer (10 μM), 12.5 μL of 2 × Phanta Max Master Mix (Vazyme), and H_2_O (add to 25 μL). PCR protocols were as follows: 95°C/4 min, 35 cycles of (95°C/30 s, 45–50°C/45 s, 72°C/1 min), and 72°C/7 min. Subsequent purification and bi‐directional Sanger sequencing are performed by the company Sangon. Overlapping fragments are merged into consensus sequences in Geneious R.11. Alignment of *COI* sequences is performed with MUSCLE algorithm (Edgar, 2004), and *16S* with MAFFT 7 (Katoh & Standley, 2013). Pairwise Kimura 2‐Parameter (K2P) distances are calculated for *Sirsoe* using the *COI* sequences (681 bp) in MEGA11 (Tamura et al., 2021). A concatenated dataset of the *COI* (681 bp) and *16S* sequences (490 bp) is obtained using SequenceMatrix 1.8 (Vaidya et al., 2011).

Phylogenetic reconstruction of *Sirsoe* is performed on the concatenated dataset using both Bayesian inference (BI) and maximum likelihood (ML) analyses. Sixty‐eight sequences downloaded from GenBank, including 21 nominal *Sirsoe* species (ingroups) and three *Nereimyra* species (outgroups), are included in the analyses. The substitution model GTR + I + G is selected for *COI* and GTR + G for *16S* in both BI and ML analyses, based on the BIC values estimated in jModelTest 2.1.10 (Darriba et al., 2012). BI analyses are performed using MrBayes v3.2 (Ronquist et al., 2012). Four Metropolis‐coupled Monte Carlo Markov chains are run for 2,000,000 generations with topologies being sampled at every 1000th generation. With the first 25% initial genealogies discarded, the remaining trees are used to generate a majority‐rule consensus tree. Posterior probability (PP) for each node is determined. Three independent runs of ML analyses are performed with the selected substitution models using IQ‐TREE 1.6.10 (Trifinopoulos et al., 2016). The supporting value for each node is determined by the ultrafast bootstrap (UBS) algorithm for 100,000 replicates. GenBank accession numbers of the sequences used in molecular analyses are provided in Table 2.

**TABLE 2:** Taxa and sequence information used in the phylogenetic study.

Taxa	Synonym	Region	Location	Habitat	Depth	COI	16s	Source
*Sirsoe ahabi* (Summers et al., 2015)	*Vrijenhoekia ahabi* Summers et al., 2015	NE Pacific	Monterey Canyon of California	Whale fall	2893	JN571876	JN571887	Summers et al. (2015)
*Sirsoe alphacrucis* Shimabukuro et al., 2019	–	Southwest Atlantic	Brazil coast	Whale fall	550–1508	MH935133, MH935134, MH935137, MH935139	MH935192–MH935194, MH935205	Shimabukuro et al. (2019)
*Sirsoe alphadelphini* Shimabukuro et al., 2019	–	Southwest Atlantic	Brazil coast	Whale fall	3285	MH935132	MH935203	Shimabukuro et al. (2019)
*Sirsoe alucia* Shimabukuro et al., 2019	–	Southwest Atlantic	Brazil coast	Whale fall	550	MH935126–MH935128	MH935158, MH935159, MH935204	Shimabukuro et al. (2019)
*Sirsoe balaenophila* lineage II	–	NE Pacific	Monterey Canyon of California	Whale fall	2893	DQ513297/JN571836	DQ513302/JN571883	Pleijel et al. (2008) and Summers et al. (2015)
		West Pacific	South China Sea	Cow fall	1604	OR133578	_	This study
*Sirsoe balaenophila* lineage I	–	NE Pacific/Southwest Atlantic	Monterey Canyon off California/Brazil coast	Whale fall	1491–4204	DQ513298, DQ513300, JN571831, JN571836, MH935087, MH935087, MH935090–MH935093	DQ513303, DQ513305/JN571884/MH935163–MH935168	Pleijel et al. (2008), Shimabukuro et al. (2019) and Summers et al. (2015)
*Sirsoe besnard* Shimabukuro et al., 2019	–	Southwest Atlantic	Brazil coast	Whale fall	3285	MH935147	MH935199	Shimabukuro et al. (2019)
*Sirsoe dalailamai* Rouse, Carvajal, et al., 2018	–	NE Pacific	Costa Rica coast	Cold seep; hydrothermal vents	1500	MG517498	MG523357	Rouse, Carvajal, et al. (2018) and Rouse, Goffredi, et al. (2018)
*Sirsoe falenothiras* (Summers et al., 2015)	*Vrijenhoekia falaenothiras* Summers et al., 2015	NE Pacific	Monterey Canyon off California	Whale fall	2893	JN571875	JN571886	Summers et al. (2015)
*Sirsoe ketea* (Summers et al., 2015)	*Vrijenhoekia ketea* Summers et al., 2015	NE Pacific	Monterey Canyon of California	Whale fall	2893	JN571838	JN571885	Summers et al. (2015)
*Sirsoe maximiano* Shimabukuro et al., 2019	–	Caribbean Sea	VonDamm field	Hydrothermal vents	2290	KJ566956	–	Plouviez et al. (2015)
		Southwest Atlantic	Brazil coast	Whale fall	1508–3358	MH935148–MH935151	MH935154–MH935157	Shimabukuro et al. (2019)
*Sirsoe methanicola* (Desbruyères & Toulmond, 1998)	*Hesiocaeca methanicola* Desbruyères & Toulmond, 1998	Atlantic	Gulf of Mexico	Cold seep	538	DQ513295	DQ442582	Desbruyères and Toulmond (1998)
*Sirsoe munki* Rouse, Carvajal, et al., 2018	–	NE Pacific	Costa Rica coast	Cold seep	1800	MG517510	MG523358	Rouse, Carvajal, et al. (2018) and Rouse, Goffredi, et al. (2018)
*Sirsoe nadir* Shimabukuro et al., 2019	–	Southwest Atlantic	Brazil coast	Whale fall	550	MH935129–MH935131	MH935200–MH935202	Shimabukuro et al. (2019)
*Sirsoe pirapuan* Shimabukuro et al., 2019	–	Southwest Atlantic	Brazil coast	Whale fall	1491–3322	MH935094–MH935097, MH935100–MH935106	MH935169–MH935178 MH935189	Shimabukuro et al. (2019)
*Sirsoe sirikos* Summers et al., 2015	–	NE Pacific	Monterey Canyon of California	Whale fall	2893	JN571829	JN571882	Summers et al. (2015)
		Southwest Atlantic	Brazil coast	Whale fall	4204	MH935152	MH935153	Shimabukuro et al. (2019)
*Sirsoe ungava* Shimabukuro et al., 2019	–	Southwest Atlantic	Brazil coast	Whale fall	1491–3358	MH935145	MH935160	Shimabukuro et al. (2019)
*Sirsoe yokosuka* Shimabukuro et al., 2019	–	Southwest Atlantic	Brazil coast	Whale fall	3322–4204	MH935141–MH935144	MH935196–MH935198	Shimabukuro et al. (2019)
*Sirsoe ypupiara* Shimabukuro et al., 2019	–	Southwest Atlantic	Brazil coast	Whale fall	3285–3358	MH935108, MH935109, MH935111, MH935114–MH935120	MH935179–MH935187, MH935190	Shimabukuro et al. (2019)
*Sirsoe* sp. A	*Vrijenhoekia* sp. A	NE Pacific	Guaymas Basin	Hydrothermal vents	1565	KP745533	KP745536	Summers et al. (2015)
*Sirsoe* sp. “BioSuOr”	–	Southwest Atlantic	Brazil coast	Whale fall	3358	MH935146	MH935161	Shimabukuro et al. (2019)
*Sirsoe polita* sp. nov.	–	WP	South China Sea	Cow fall	655	OR126114 –OR126116	OR129671–OR129673	This study
*Sirsoe nanhaiensis* sp. nov.	–	WP	South China Sea	Cow fall	1602	OR126101–OR126113, OR126129	OR129656	This study
*Sirsoe feitiana* sp. nov.	–	WP	South China Sea	Cow fall	3402	OR126117–OR126128, OR129992, OR129993	OR129662–OR129665	This study
*Nereimyra aphroditoides* (O. Fabricius, 1780)	*Nereis aphroditioides* Fabricius, 1780	–	–	–	–	JF317198–JF317200	JF317211–JF317214	–
*Nereimyra punctata* (Müller, 1788)	*Nereis punctata* Müller, 1788	–	–	–	–	DQ442566	DQ442577	–
*Nereimyra woodsholea* (Hartman, 1965)	*Neopodarke woodsholea* Hartman, 1965	–	–	–	–	AY644805	JF317215	–

### 2.5 Detection of *Sirsoe* with metabarcoding analyses

To examine if any other *Sirsoe* species in addition to the sampled specimens is present at each location, a surface layer (0–4 cm) of sediment is collected using pushcore sampler from each of the three cow falls. Only one sediment sample was obtained from each of the 655‐ and 1604‐m site, while two separate sediment samples were collected at the 3402 m, both of which are treated as reciprocal “biological replicates” to address the concern about amplification errors and confirm the presence of each detected OTU at this site. For each sample, about 2–5 g sediment is used for total DNA extraction and then prepared for subsequent metabarcoding sequencing. Short fragments of *COI* are amplified using primers mlCOIintF (5′‐GGWACWGGWTGAAWACWGGWTGAACCYCC‐3′) (Leray et al., 2013) and jgHCO2198 (5′‐TAIACYTCIGGRTGICCRAARAAYCA‐3′) (Geller et al., 2013). The Illumina high‐throughput sequencing of amplicons is performed on Illumina NovaSeq platform at Mingke Biotechnology Co., Ltd. Raw data filtration is performed using Trimmomatic v0.33 and cutadapt 1.9.1, and the resulting high‐quality reads are assembled with FLASH v1.2.7 (Magoč & Salzberg, 2011) and then processed in UCHIME v4.2 (Edgar et al., 2011) to remove chimeric sequences. As 3% divergence of *COI* is the threshold value for inter‐OTU delineation commonly accepted in metabarcoding research (Li et al., 2022), and also the lowest value of *COI* divergence observed between nominal *Sirsoe* species (see below in the “Molecular and phylogenetic analyses”), sequences with ≥3% divergence from each other and nominal species are assigned to distinct OTUs using USEARCH (version 10 http://drive5.com/uparse/). The OTUs, which represent less than 0.01% of the total reads, were excluded in subsequent analyses except when they are present in both replicates. Taxonomic annotation of each OTU is carried out by querying GenBank using UCLUST v1.2.22q. Sequences temporarily assigned to *Sirsoe* (including *Vrijenhoekia*) are picked out and integrated into a dataset including all *COI* sequences of the two genera (*Sirsoe* and *Vrijenhoekia*) deposited in the GenBank as well as those newly obtained in the present study. K2P distance is calculated for each lineage pair based on *COI* dataset (about 300 bp) in MEGA11 (Tamura et al., 2021). Both ML and BI trees are reconstructed using the same methods mentioned above.

## 3 RESULTS

### 3.1 Molecular and phylogenetic analysis

**FIGURE 2:**
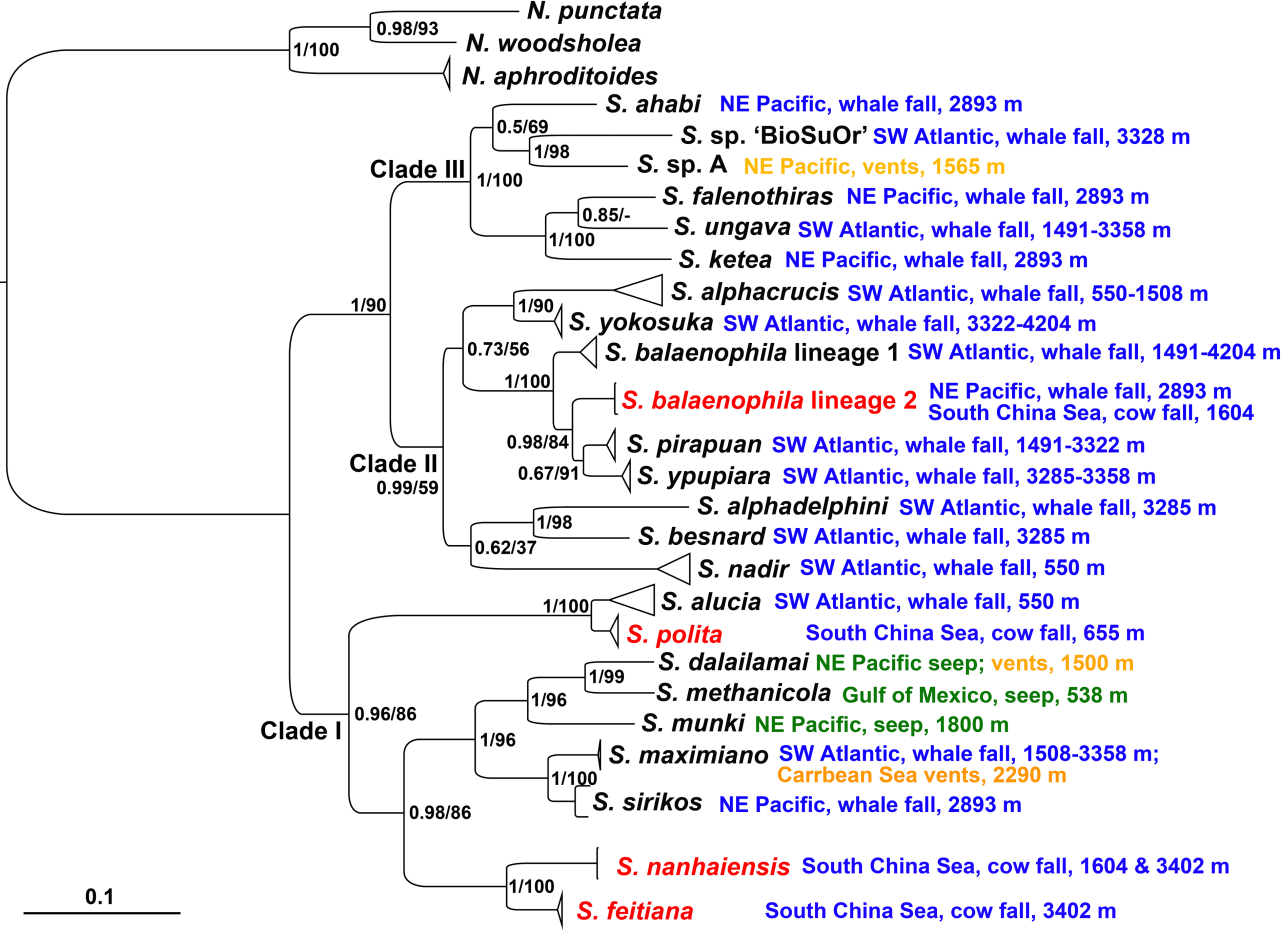
Phylogenetic reconstruction of global *Sirsoe* species using concatenated dataset of *COI* and *16S* genes. Support values are given as PP/UBS value. The species names of the SCS records are in red color. Geographic range and habitat type of each species are provided behind its name, with those from mammal falls colored in blue, those from hydrothermal vents in orange, and those from cold seeps in green.

Both BI and ML analyses based on the concatenated dataset (681‐bp *COI* and 490‐bp *16S*) reveal a three‐clade topology similar to that in Shimabukuro et al. (2019), except for some interior nodes (Figure 2). *Sirsoe* is monophyletic when synonymized with *Vrijenhoekia* and divided into three clades. Clade I receive high support in both BI and ML analyses (PP/UBS = 1/87), with all its internal nodes well resolved (Figure 2). It is sister to a well supported branch (PP/UBS = 1/90) formed by clade II (PP/UBS = 1/100) and III (PP/UBS = 1/66) (Figure 2). Although the three clades do not cluster with either geographic region or habitat type, clade I and III cluster species are mainly from the Pacific region (including the NE Pacific and SCS), while clade II is mainly composed of species from the SW Atlantic whale falls with only one exception (*S. balaenophila* lineage II) (Figure 2). Three seep species form a monophyletic subclade in clade I and the three vent habitants are scattered in clade I and II (Figure 2).

Seventy‐four SCS individuals are placed in clade I, forming three well‐supported distinct lineages (Figure 2), while an incomplete individual forms one lineage with *S. balaenophila* lineage II in clade II. Compared with the relatively high inter‐lineage K2P distances (3.3%–29.6%) (Table 3), the extremely low intra‐lineage K2P distances (always less than 1%) of the three new SCS lineages indicate that the SCS individuals placed in clade I represent three species new to science, which are also morphologically distinctive and described as *Sirsoe polita* sp. nov., *Sirsoe nanhaiensis* sp. nov., and *S. feitiana* sp. nov., respectively below. The inter‐specific K2P distances calculated between each of the three SCS species and other congeners range 3.4%–29.4% for *Sirsoe polita* sp. nov., 12.6%–28.2% for *S. nanhaiensis* sp. nov., and 12.6%–27.9% for *S. feitiana* sp. nov. (Table 3). For the remaining SCS individual in clade II, it is impossible to perform morphological description due to the poor condition, and is genetically assigned to *S. balaenophila* lineage II based on the low K2P distance value (<1%).

**TABLE 3:** Pairwise K2P distances between and within lineages.

Taxa/OTU	1	2	3	4	5	6	7	8	9	10	11	12	13	14	15	16
1. OTU1 (OR136433)	–															
2. OTU36 (OR136434)	20.8	–														
3. OTU82 (OR136435)	22.3	27.1	–													
4. OTU296 (OR136436)	4.3	25.9	27.1	–												
5. OTU335 (OR136437)	3.3	25.1	26.7	4.6	–											
6. OTU416 (OR136431)	21.7	4.0	25.6	26.8	26.0	–										
7. OTU227 (OR136430)	20.7	24.4	9.0	25.4	25.0	23.9	–									
8. OTU462 (OR136432)	24.2	20.2	24.6	28.5	28.1	21.5	24.7	–								
9. OTU810 (OR136438)	3.9	25.0	26.6	4.3	4.3	25.9	24.5	28.1	–							
10. *S. ahabi*	21.9	20.1	20.3	27.6	26.3	21.0	20.3	26.1	26.7	–						
11. *S. alphacrucis*	25.6	24.8	17.5	30.8	30.4	24.4	18.0	29.2	29.8	19.9	0.2					
12. *S. alphadelphinidel*	31.3	28.3	19.0	36.9	36.5	29.8	22.2	31.0	36.4	21.5	22.0	–				
13. *S. alucia*	22.5	21.1	24.4	27.1	26.7	22.6	25.3	3.2	26.7	25.5	26.7	27.6	0.0			
14. *S. balaenophila* lineage I	21.2	23.0	8.7	26.2	25.7	23.0	7.6	26.0	25.7	18.0	16.7	21.2	23.5	1.3		
15. *S. balaenophila* lineage II	21.6	24.5	9.3	26.3	25.9	23.7	0.3	25.2	25.4	17.3	18.3	20.6	25.8	8.2	0.3	
16. *S. besnard*	26.3	25.2	21.0	32.1	31.2	26.1	20.6	26.3	31.1	19.7	20.3	18.5	24.9	18.7	18.2	–
17. *S*. “BioSuOr”	30.7	27.3	25.8	37.1	36.1	25.5	24.3	29.6	35.3	18.4	20.5	23.8	25.6	18.9	20.4	22.3
18. *S. dalailamai*	20.9	16.2	25.5	26.1	25.2	14.6	22.0	24.7	25.7	23.7	21.2	25.6	22.4	22.9	22.4	21.2
19. *S. falenothiras*	26.0	21.2	21.9	31.5	30.6	20.2	22.1	28.7	30.5	18.5	19.4	23.0	27.8	19.7	20.5	22.3
20. *S. ketea*	24.5	23.3	20.3	29.8	28.9	23.3	19.0	27.7	28.3	20.4	23.5	22.7	27.8	20.0	20.8	23.5
21. *S. maximiano*	21.9	15.5	25.5	27.4	26.5	15.1	24.5	22.6	25.9	23.3	21.9	28.3	23.9	23.4	25.1	24.1
22. *S. methanicola*	24.3	19.0	28.0	29.2	28.8	18.6	25.6	25.8	28.3	26.8	25.8	29.6	25.3	24.1	24.5	25.8
23. *S. munki*	22.2	13.9	28.1	27.9	26.5	11.1	29.3	27.0	26.9	26.8	22.9	29.1	26.4	24.4	24.6	23.8
24. *S. nadir*	31.1	27.7	25.6	36.7	36.3	27.9	24.6	27.6	35.5	22.1	23.2	21.1	27.1	20.4	20.9	22.0
25. *S. pirapuan*	22.4	26.4	8.4	27.6	27.1	24.7	6.1	29.0	27.1	18.5	18.1	21.2	26.3	7.1	6.4	19.1
26. *S. sirikos*	23.0	16.9	26.8	28.9	27.7	15.8	25.0	24.6	27.4	23.4	20.8	27.4	25.4	22.1	22.5	22.0
27. *S*. sp.A	29.7	24.6	24.3	34.8	34.1	24.1	22.9	28.9	33.9	16.9	23.1	21.4	26.9	19.3	20.8	23.7
28. *S. ungava*	24.9	21.2	21.2	30.6	29.7	19.7	19.9	27.5	29.6	18.9	16.8	23.6	27.6	18.1	18.5	21.6
29. *S. yokosuka*	25.7	25.4	14.7	31.1	30.7	24.7	17.2	27.8	30.0	17.2	12.0	19.8	24.2	14.5	16.0	18.7
30. *S. ypupiara*	22.4	25.4	4.5	27.9	27.0	24.3	7.6	26.5	27.5	19.0	19.5	21.0	26.0	8.3	8.3	19.0
31. *S. nanhaiensis*	11.4	17.7	25.0	16.9	15.6	17.2	21.6	23.2	16.5	20.8	23.4	28.2	24.2	21.4	23.2	22.6
32. *S. feitiana*	0.1	20.6	22.4	4.5	3.5	21.5	20.8	24.2	4.2	20.8	23.4	27.9	20.8	19.7	20.5	23.3
33. *S. polita*	23.7	19.8	24.8	28.2	27.8	21.1	25.1	0.4	27.7	24.5	28.2	29.4	3.4	24.5	24.8	24.8

Taxa/OTU	17	18	19	20	21	22	23	24	25	26	27	28	29	30	31	32	33
1. OTU1																	
2. OTU36																	
3. OTU82																	
4. OTU296																	
5. OTU335																	
6. OTU416																	
7. OTU227																	
8. OTU462																	
9. OTU810																	
10. *S. ahabi*																	
11. *S. alphacrucis*																	
12. *S. alphadelphinidel*																	
13. *S. alucia*																	
14. *S. balaenophila* lineage I																	
15. *S. balaenophila* lineage II																	
16. *S. besnard*																	
17. *S*. “BioSuOr”	–																
18. *S. dalailamai*	23.9	–															
19. *S. falenothiras*	17.5	22.9	–														
20. *S. ketea*	21.6	25.4	16.0	–													
21. *S. maximiano*	23.0	18.0	22.5	24.7	0.1												
22. *S. methanicola*	25.0	11.4	22.8	23.7	17.8	–											
23. *S. munki*	26.5	15.1	24.5	25.9	17.2	14.9	–										
24. *S. nadir*	22.3	24.4	22.9	23.2	25.6	25.9	28.5	0.9									
25. *S. pirapuan*	18.7	23.1	21.3	21.5	23.1	24.3	23.0	20.6	0.3								
26. *S. sirikos*	24.6	16.9	21.6	26.1	8.2	17.4	15.9	23.7	22.3	3.0							
27. *S*. sp.A	17.2	23.5	18.6	20.6	25.0	26.8	27.7	22.3	19.1	24.3	–						
28. *S. ungava*	19.7	20.3	11.6	16.4	23.6	23.0	24.0	20.1	18.5	22.1	18.9	–					
29. *S. yokosuka*	18.4	22.2	18.8	20.5	24.0	27.8	26.5	21.0	16.0	23.6	20.1	16.4	0.5				
30. *S. ypupiara*	20.5	25.2	21.5	21.5	25.4	25.4	25.4	23.0	6.4	23.9	21.3	19.8	16.5	0.4			
31. *S. nanhaiensis*	23.1	17.3	23.6	26.1	17.9	21.9	21.0	23.3	22.9	17.9	21.9	21.8	22.0	24.1	0.2		
32. *S. feitiana*	24.5	16.9	23.6	24.1	18.2	19.2	19.8	24.5	20.2	18.2	23.4	22.2	23.0	21.4	12.6	0.3	
33. *S. polita*	26.2	22.2	26.4	27.1	23.6	24.6	25.3	26.6	26.2	25.1	25.4	27.3	25.7	25.6	22.6	20.9	0.5

*Note*: Numbers are shown in percentage (%). Accession numbers of OTU sequences are provided.

*Sirsoe polita* sp. nov., collected only from the 655‐m site, is recovered with high support (PP/UBS = 1/100) as sister to *S. alucia* from the same depth range (~550 m) in the SW Atlantic, resulting in a well‐supported subclade deeply divergent from others in the clade (Figure 2). Interestingly, they form a sister species pair with the shallowest divergence (K2P distance 3.4%) but the widest geographic range (from SW Atlantic to SCS in the West Pacific). The two deep SCS species, *Sirsoe nanhaiensis* sp. nov. (collected from both the 1604‐ and 3402‐m sites) and *S. feitiana* sp. nov. (collected from the 3402‐m site), are recovered as sisters to each other (PP/UBS = 1/100, K2P distance 12.6%), forming a lineage distinct from all other congeners in clade I (Figure 2).

### 3.2 *Sirsoe* OTUs detected with metabarcoding

**FIGURE 3:**
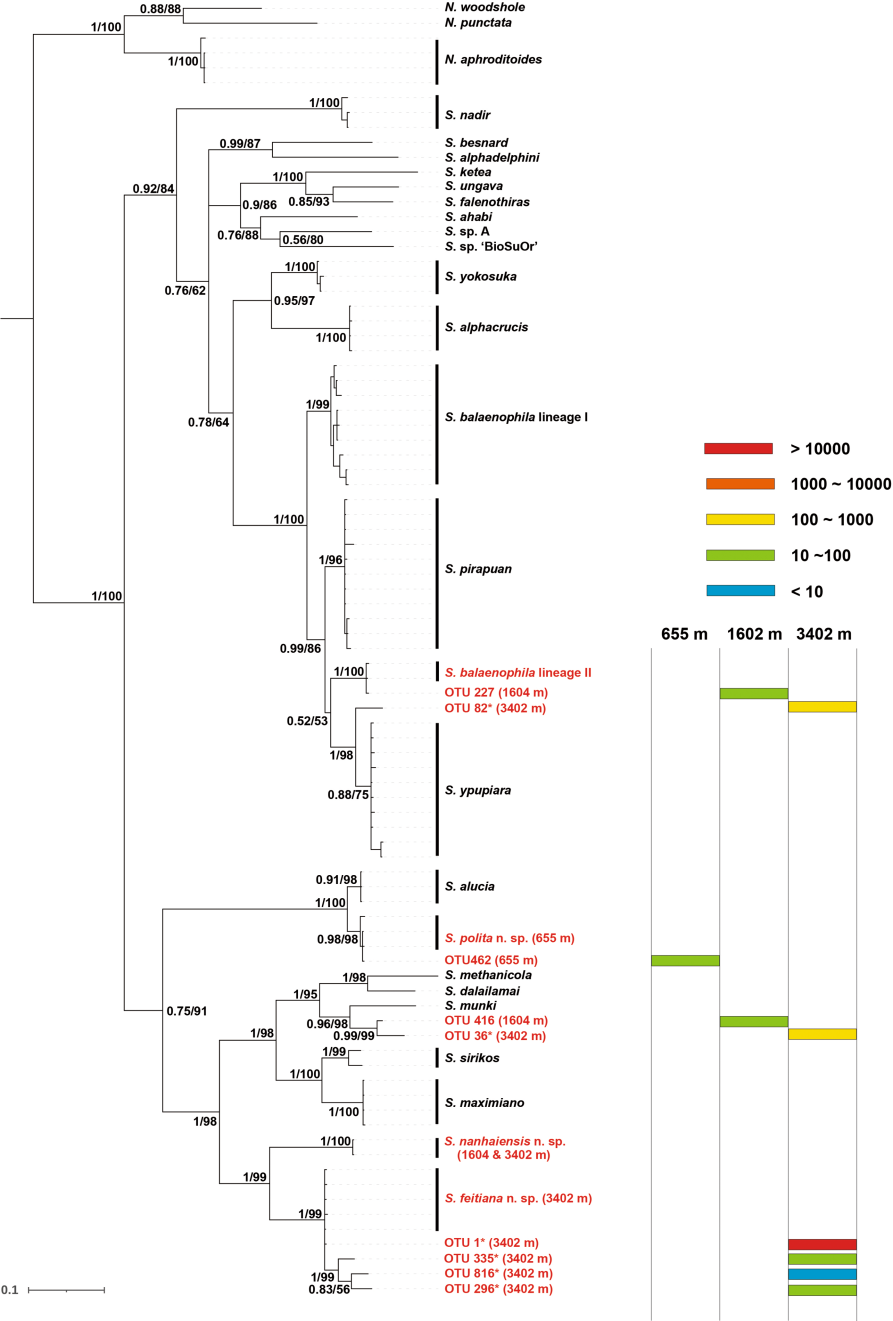
Phylogenetic reconstruction of *Sirsoe* species/OTUs based on partial *COI* sequences. Support values are given as PP/UBS value. The SCS records are colored in red. Read number of each OTU are scaled in colors on the right. OTUs marked with “*” are detected in both replicates at the 3402‐m site.

Illumina sequencing of *COI* amplicons from three cow‐fall sediment samples reveal nine OTUs belonging to *Sirsoe*, with three assigned to nominal species (similarity 97%–100%) and six treated as distinct OTUs from other species/OTUs (similarity 87%–97%). The addition of OTUs does not change the tree topology significantly (Figure 3). In total, one, two, and six OTUs are recovered at the 655‐, 1604‐, and 3402‐m cow falls, respectively, with each OTU found at single site (Figure 3). All the six OTUs from the 3402‐m stie are detected, although varied slightly in read numbers, in both replicates. OTU 462 (read number: 21), detected at the 655‐m cow fall, is assigned to *S. polita* sp. nov. (100% similarity) (Figure 3). In the 1604‐m cow fall, OTU 227 (read number: 79) is assigned to *S. balaenophila* lineage II (99.7% similarity) reported from the East Pacific, and OTU 416 (read number: 29) shows the highest similarity to OTU 36 (read numbers: 577 and 390) found in the 3402‐m cow fall (96.1% similarity), both of which form a species pair sister to *S. munki* (87% similarity) (Figure 3). However, we fail to detect *S. nanhaiensis* sp. nov. with metabarcoding in both 1604‐ and 3402‐m cow falls. In the 3402‐m cow fall, the OTU1, assigned to *S. feitiana* sp. nov. (100% similarity), is detected in high abundance in both replicates (>10,000 reads) (Figure 3); OTU 82 (read numbers 154 and 62) is recovered to be sister to *S. ypupiara* (95.6% similarity); three additional OTUs (296, 335, 816), all of which are detected in low numbers in both replicates, form a distinct subclade nested within haplotypes of *S. feitiana*/OTU1, with which they show genetic divergence at the lower range of interspecific distance (K2P distance 3.3%–4.7%) (Figure 3).

### 3.3 Taxonomy

#### Class: Polychaeta

#### Order: Phyllodocida

#### Family: Hesionidae Grube, 1850

#### Genus: *Sirsoe* Pleijel, 1998

#### ***Sirsoe polita*** sp. nov. (Figure 4)

##### ZooBank registration

rn:lsid:zoobank.org:act:6AA01C81‐21E1‐4F96‐AC90‐841BC7F3157E

##### Diagnosis

Tough and small‐sized *Sirsoe*, about 10 mm in length. Proboscis smooth, with 10 terminal papillae, foliaceous.

##### Type locality

In a cow fall, Zhongnan seamount, South China Sea, 115.423°E, 13.942°N, 655 m depth.

##### Type materials

Holotype (SY400001) in 100% ethanol, 7.4 mm in length and 1.7 mm in width (without parapodia), 21 segments, a cow fall deployed on Zhongnan seamount, South China Sea, 115.423°E, 13.942°N, 655 m deep (station “ZS4”), R/V *Tansuo2* cruise TS2‐8, by a suction sampler mounted on HOV *Shenhaiyongshi*, dive SY400, July 13, 2021. Paratypes (SY400005, SY400014, SY400031, SY400032, SY400033, SY400034, SY400035, SY400036, SY400037, SY400038, and SY400040), 100% ethanol, 6.4–13.2 mm in length and 1.1–2.5 in width (without parapodia), 21–26 segments, same as holotype.

##### Description

**FIGURE 4:**
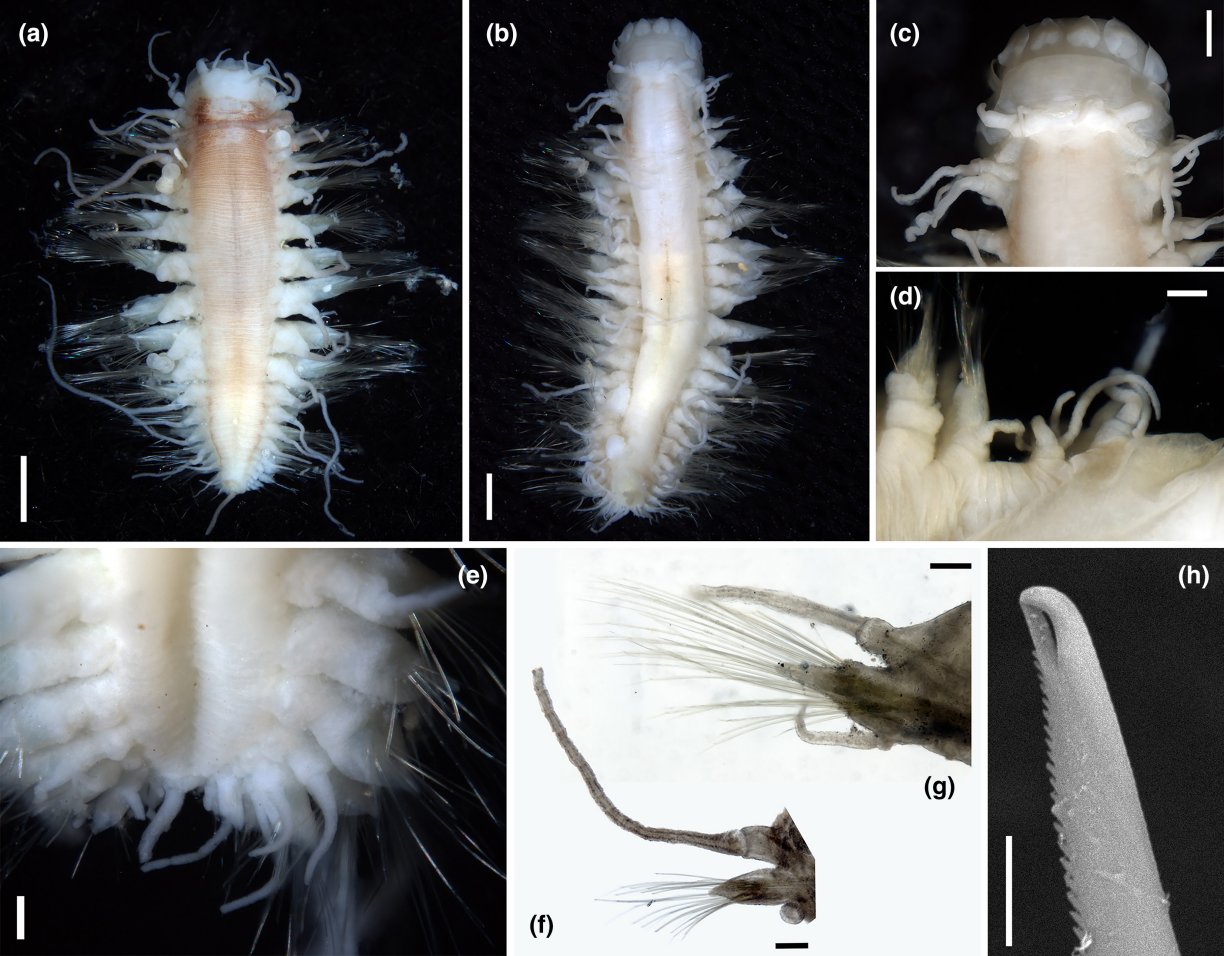
*Sirsoe polita* sp. nov. (a) dorsal view, holotype (SY400001); (b) dorsal view, paratype (SY400031); (c) anterior end, dorsal view, paratype (SY400031); (d) first four segments, right side, ventral view, paratype (SY400031); (e) posterior end, ventral view, paratype (SY400005); (f) parapodia on one posterior segment, posterior view, paratype (SY400005); (g) parapodia on segment 11, anterior view, paratype (SY400040); (h) distal part of neurochaetae on segment 8, holotype (SY400001). Scales: (a, b) 1 mm; (c) 0.5 mm; (d–g) 0.2 mm; (h) 5 μm.

Body stout, tapering in posterior half of body; preserved specimens pale white except for slightly brownish pigmentation on dorsum, especially on the first three segments (Figure 4a,b). Prostomium trapezoid, wider than long, slightly bilobed anteriorly; basally forming a ridge extending laterally to the first segment when proboscis everted (Figure 4a–c). Frontal tubercle not observed; eyes absent; nuchal organs absent or indistinct. Palp biarticulated, with cylindrical palpophores and conical and slightly shorter palpostyles (Figure 4c). Paired antennae filiform, approximately equal in length to palp; median antennae absent (Figure 4a,c). Glandular lip pads absent. Proboscis with 10 terminal papillae, foliaceous, without ciliation ornament among each other (Figure 4b,c). First three segments fused. All dorsal cirri, long; first three segments with enlongated ventral cirri and distinct cirrophore (Figure 4d); ventral cirri on following segments short, equal in length to or longer than acicular lob of parapodia, cirrophore indistinct (Figure 4e).

First two segments achaetous. Neuropodia and neurochaetae begin on segment three (Figure 1d). Parapodia sub‐biramous, with pointed triangular prechaetal lobe and shorter rounded postchaetal lobe (Figure 4f,g). Neurochaetae compound; supra‐acicular and sub‐acicular neurochaetae with shorter blades than middle ones. Blades distally curved, unidentate, with sub‐distal prolongation and finely serrated cutting edges (Figure 4h). Pygidial cirri one pair, slender and terminal (Figure 4e).

##### Distribution

Currently known only from a cow fall on Zhongnan seamount in the SCSat a depth of 655 m.

##### Etymology

“*polita*”, meaning smoothed in Latin, refers to the smoothed proboscis without ciliation between foliaceous papillae. Therefore, the suggested formal Chinese name is “光洁神女虫”.

##### Remarks

*Sirsoe polita* sp. nov. resembles *S. alucia* in most characters, except for the proboscis, which bears significant ciliation between terminal papillae in the latter species. The two species also have a *COI* divergence (3%–4%) with each other much lower than with other congeners, which is the lowest interspecific *COI* divergence value in this genus.

#### ***Sirsoe nanhaiensis*** sp. nov. (Figure 5)

##### ZooBank registration

urn:lsid:zoobank.org:act:8DF15C71‐E93E‐4237‐ABC8‐974B78BD7254

##### Diagnosis

Large‐sized *Sirsoe*. Body soft, semi‐transparent. Cirriphore on the first segment indistinct. Dorsal cirriphore on following segments prominent, long, and swollen. Neuropodia well developed, with long pointed dorsal projections.

##### Type locality

In a cow fall, Zhongnan seamount, South China Sea, 115.430°E, 13.915°N, 1604 m depth.

##### Type materials

Holotype (SY396P01) in 100% ethanol, 43 mm in length and 3.9 mm in width (without parapodia), 44 segments, a cow fall deployed on the mountainside of Zhongnan seamount, South China Sea, 115.430°E, 13.915°N, 1604 m deep (station “ZS2”), R/V *Tansuo2* cruise TS2‐8, collected together with a cow bone by the 7‐function manipulator of HOV *Shenhaiyongshi*, dive SY400, July 09, 2021. Paratypes (SY3960001, SY3960002, SY396CB2, SY396P003, SY396P005, SY396P006, SY396P007, SY396BP01, and SY396BP04), 100% ethanol, 18.9–27.1 mm in length and 1.4–2.8 in width (without parapodia), 27–36 segments, same as holotype.

##### Description

**FIGURE 5:**
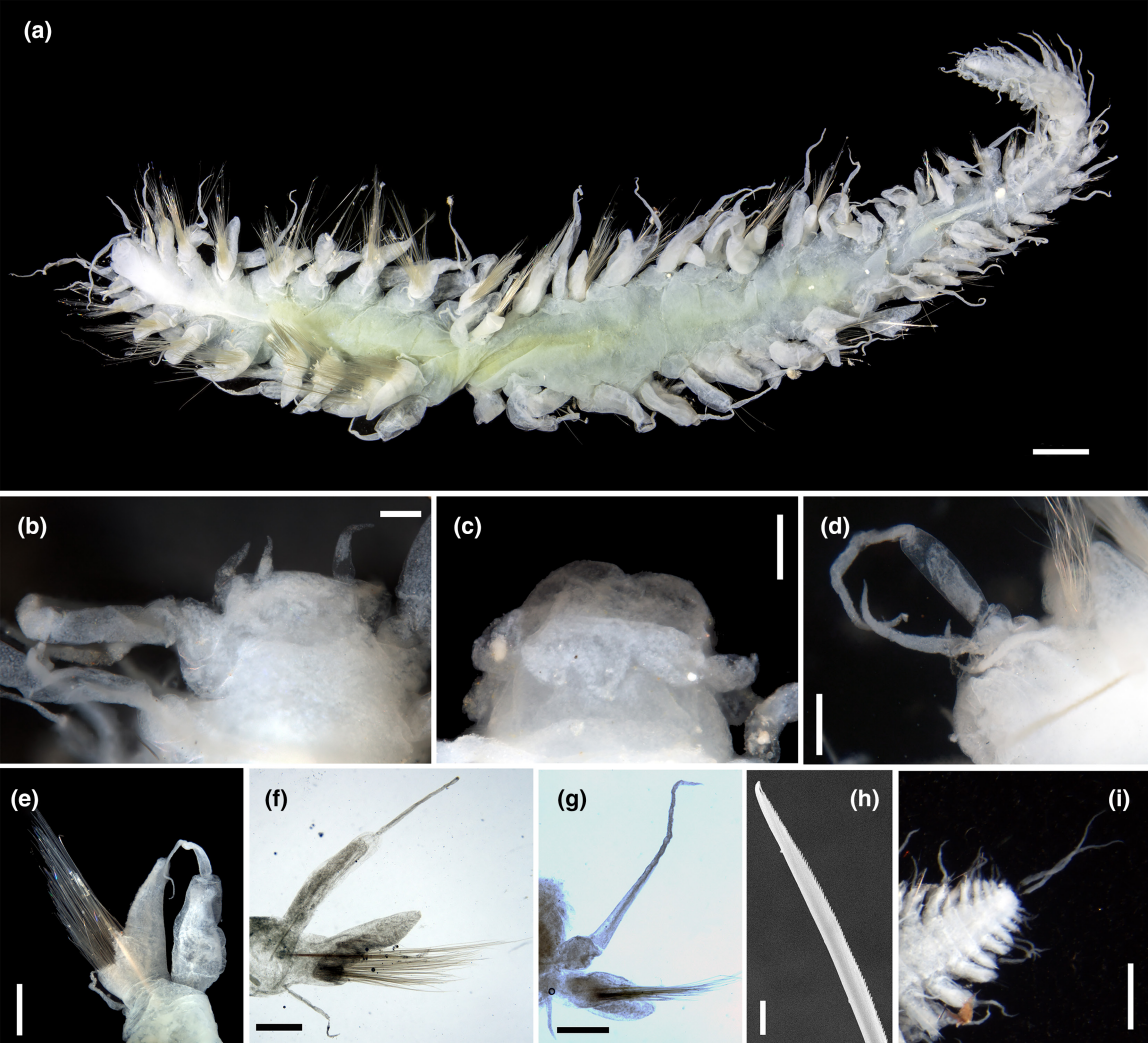
*Sirsoe nanhaiensis* sp. nov. (a) complete specimen, ventral view of anterior part and dorsal view of posterior part, holotype (SY396P01); (b) anterior end, dorsal view, paratype (SY396P03); (c) anterior end, dorsal view, paratype (SY396P06); (d) anterior end, ventral view, paratype (SY396P02); (e) parapodia on segment 10, anterior view, holotype (SY396P01); (f) parapodia on segment 11, posterior view, holotype (SY396P01); (g) parapodia in posterior region, paratype (SY396CB2); (h) distal part of neurochaetae on segment 11; (i) posterior end, ventral view, paratype (SY396P05). Scales: (a) 2 mm; (b, c) 0.2 mm; (d, g) 0.5 mm; (e, f, i) 1 mm; (h) 10 μm.

Body soft, tapering posteriorly; preserved specimens pale white and semi‐transparent; some individuals with light green midgut (Figure 5a). Prostomium rectangular, wider than long (Figure 5b). Frontal tubercle not observed; eyes absent; nuchal organs absent or indistinct. Palp biarticulated; palpophores short, cylindrical; palpostyles conical, longer than palpophores (Figure 5b). Paired antennae conical, as stout and long as palp; median antennae absent (Figure 5b). Glandular lip pads absent. Proboscis surrounded by 10 terminal papillae, foliaceous, smooth (Figure 5c). First segment achaetous; dorsal cirri long, with short indistinct cirrophore; ventral cirri filiform, shorter than dorsal cirri, with indistinct cirrophore (Figure 5d). Dorsal cirrophores on the following segments long, swollen, cylindrical, but globular and less prominent in posterior segments; dorsal cirrostyles slender, longer than cirrophores, extending beyond neurochaetae (Figure 5a,e–g). Ventral cirri short on the following segments, slender, with indistinct cirrophores (Figure 5e–g).

Parapodia sub‐biramous (Figure 5e–g). Notopodia reduced and fused with dorsal cirriphore, bearing 1–2 acicula (Figure 5e–g). Neuropodia well‐developed, with triangular prechaetal acicular lobes and shorter rounded postchaetal lobes; dorsal projections on neuropodia pointed, much longer than prechaetal acicular lobes (Figure 5e–g). Neurochaetae numerous, compound, forming fan‐shaped bundles, with supra‐acicular neurochaetae longer than sub‐acicular ones. Supra‐acicular and sub‐acicular neurochaetae with shorter blades than middle ones (Figure 5f,g). Blades distally curved, unidentate, with sub‐distal prolongation and finely serrated cutting edges (Figure 5h). Pygidial cirri one pair, long, slender and terminal (Figure 5i).

##### Distribution

Currently known only from two cow falls on the Zhongnan seamount in the South China Sea at a depth of 1604 and 3402 m, respectively.

##### Etymology

“nanhai”, referring to the SCS in Chinese, type location of the new species. The suggested formal Chinese name is “南海神女虫”.

#### ***Sirsoe feitiana*** sp. nov. (Figure 6)

##### ZooBank registration

urn:lsid:zoobank.org:act:F0E3798D‐149B‐4C91‐98D0‐E2B0226C29A5

##### Diagnosis

Large‐sized *Sirsoe*. Body soft, semi‐transparent. Dorsal and ventral cirri on first segment with short but distinct cylindrical cirriphore. Dorsal cirriphore on following segments prominent, long, swollen. Neuropodia well‐developed, with long pointed dorsal projections.

##### Type locality

In a cow fall, Zhongnan seamount, South China Sea, 115.385°E, 13.861°N, 3402 m depth.

##### Type materials

Holotype (SY398007) in 100% ethanol, 25 mm in length and 2.9 mm in width (without parapodia), 38 segments, a cow fall deployed near the bottom of Zhongnan seamount, South China Sea, 115.385°E, 13.861°N, 3402 m deep (station “ZS3”), R/V *Tansuo2* cruise TS2‐8, collected in pushcore by HOV *Shenhaiyongshi*, dive SY398, July 11, 2021. Paratypes (SY398001, SY39806, SY398012, SY398014, SY398017, SY398018, SY398020, SY398024, SY398027, and SY398028), 100% ethanol, 11.1–13.7 mm in length and 0.8–1.6 in width (without parapodia), 25–35 segments, same as holotype.

##### Description

**FIGURE 6:**
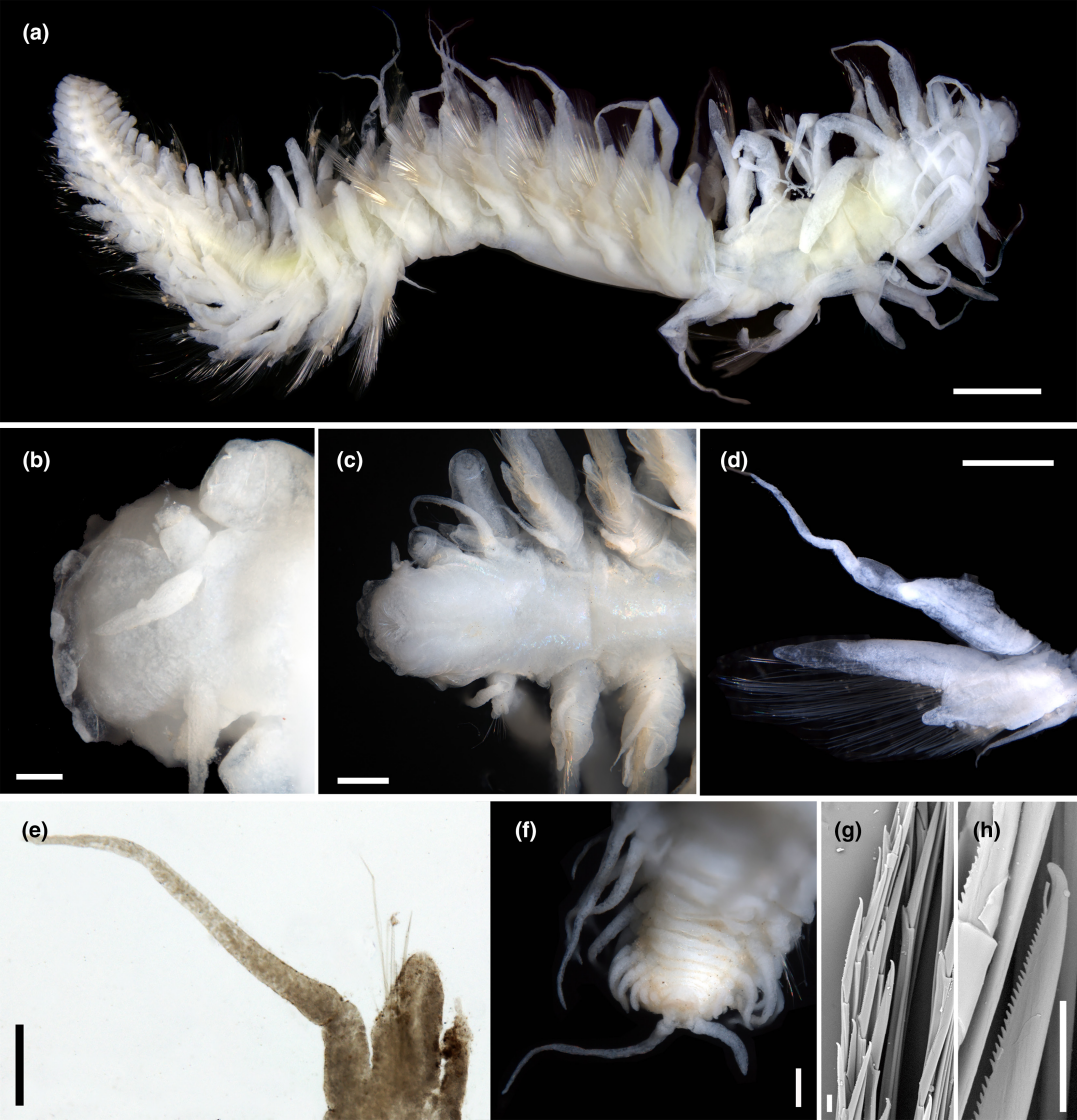
*Sirsoe feitiana* sp. nov. (a) complete specimen, dorsal views of anterior and posterior part and lateral view of middle part, holotype (SY398007); (b) anterior end, dorsal view, holotype (SY398007); (c) anterior end, ventral view, holotype (SY398007); (d) parapodia on segment 9, anterior view, holotype (SY398007); (e) parapodia on segment 24, posterior view, paratype (SY398006); (f) posterior end, dorsal view, paratype (SY398006); (g) neurochaetae on segment 10, holotype (SY398007); (h) details of distal part of neurochaetae on segment 10, holotype (SY398007). Scales: (a) 2 mm; (b, e, f) 0.2 mm; (c) 0.5 mm; (d) 1 mm; (g, h) 20 μm.

Body soft, tapering posteriorly; preserved specimens pale white, sometimes with light green midgut (Figure 6a). Prostomium rounded rectangular, wider than long (Figure 6b). Frontal tubercle not observed; eyes absent; nuchal organs absent or indistinct. Palp biarticulated with short cylindrical palpophores and conical and slightly longer palpostyles (Figure 6b,c). Paired antennae filiform, approximately equal in length to palp; median antennae absent (Figure 6b). Glandular lip pads absent. Proboscis with 10 terminal papillae, half‐rounded, without ciliation (Figure 6b). First segment achaetous; dorsal cirri long, with short cylindrical cirriphore; ventral cirri filiform, shorter than dorsal cirri, with short cylindrical cirriphore (Figure 6c). Dorsal cirri on following segments long; cirrophores long, prominent, swollen, cylindrical, slightly shorter than parapodia, but globular and less prominent in posterior segments; styles slender, longer than cirrophores, extending beyond neuropodial chaetae (Figure 6d–f). Ventral cirri on the first two segments elongated, with distinct cirrophores and much longer than the ones on following segments (Figure 6c,d).

Parapodia sub‐biramous, with triangular prechaetal acicular lobes, shorter rounded postchaetal lobes and very long pointed dorsal projections (Figure 6d,e). Neurochaetae numerous, compound, forming fan‐shaped bundles, with supra‐acicular neurochaetae longer than sub‐acicular ones (Figure 6d,g). Supra‐acicular and sub‐acicular neurochaetae with shorter blades than middle ones. Blades distally curved, unidentate, with sub‐distal prolongation and finely serrated cutting edges (Figure 6g,h). Pygidial cirri one pair, slender and terminal (Figure 6f).

##### Distribution

Currently known only from a cow fall at the mountain foot of Zhongnan seamount in the SCS at a depth about 3400 m.

##### Etymology

“feitian”, meaning “flying apsara” in Chinese. In the famous murals in Yungang, Longmen, and Dunhuang Grottoes in China, they are featured by their long ribbon fluttering elegantly and beautifully. The new species also have long and elegant cirrophores which resemble Feitian's ribbon, giving a sense of flying or drifting. The suggested formal Chinese name is “飞天神女虫”.

##### Remarks

Both *S. nanhaiensis* sp. nov. and *S. feitiana* sp. nov. differ from other congeners in their long dorsal cirriphore along the body, and well‐developed neuropodia with long pointed dorsal projections, while it is not easy to separate the two new species from each other by morphological features. The two new species are almost identical to each other, except for some minute differences: (1) the short cylindrical cirriphore on the first segment in *S. feitiana* sp. nov. contrasts with the indistinct ones in *S. nanhaiensis* sp. nov., and (2) two rows of spines on neurochaetae in the former are also different from one row of spines in the latter. *S. feitiana* sp. nov. is exclusively found in collections from the 3402‐m site, while *S. nanhaiensis* sp. nov. is mainly collected from the 1604‐m cow fall with five individuals from the 3402‐m cow fall. Due to the high morphological similarity between the two species, these five individuals could have been misidentified as *S. feitiana* sp. nov. in case we did not perform barcoding analyses on almost all collected specimens, including those incomplete pieces. This strongly prove the importance of DNA‐based methods in distinguishing deep‐sea cryptic species (Wang et al., 2020).

## 4 DISCUSSION

With the experimentally deployed cow falls, we investigate the biodiversity of *Sirsoe* species to gain insight into the diversity and biogeography of the deep SCS basin. A total of four *Sirsoe* species are sampled from the three cow falls, with only 1–2 representatives recovered in each site, showing much lower species richness relative to that at whale falls in the Southwest Atlantic and the NE Pacific (2–6 species per whale fall) (Shimabukuro et al., 2019). Although carcasses of cows and whales may vary in many aspects (i.e., size, weight, and amount of bone lipids), we doubt that if differences in substrate type (cows vs. whales) can be evoked to explain such variations in hesionid diversity. Rouse, Goffredi, et al. (2018) found no obvious difference among *Osedax* species colonizing bones of different vertebrate origins, and we speculate a low selectivity on substrate type for opportunists (e.g., hesionids) in a nutrient‐limited environment.

Only 1 out of 75 individuals is genetically identified as *S. balaenophila* lineage II, which indicates that some rare species may be easily ignored based only on ordinary sampling and analytic approach (such as specimen collection and morphological examination). To explore “hidden diversity” consisting of either rare or small species, we use metabarcoding analyses to check if there are *Sirsoe* species occurring at the three cow falls in addition to those sampled ones. Nine OTUs assigned to *Sirsoe* are recovered by metabarcoding, which increases the *Sirsoe* diversity to 10 species/OTUs, with an average of one to seven revealed at each site. Three out of the four collected species are detected by metabarcoding, including the rare species *S. polita* and *S. balaenophila* lineage II, and the failure to detect *S. hainanensis* at the two deep sites may be due to poor primer fit. All the six 3402‐m OTUs are detected in both replicates, verifying their presences at the 3402‐m cow fall, although in low abundance (<100) in some cases. This result indicates that *Sirsoe* diversity in the SCS might be much higher than estimated with an ordinary sampling approach. The failure of sampling of the OTUs detected by metabarcoding may be due to either their low abundance present in the community, or high heterogeneity at a fine temporal–spatial scale (Rouse, Goffredi, et al., 2018; Smith & Baco, 2003; Smith et al., 2015).

The semi‐enclosed nature may give rise to a hypothesis of a high level of endemism in the deep SCS basin as what occurred in the Mediterranean Sea (Danovaro et al., 2010). The LS has been proposed as a boundary between the SCS and neighboring provinces (Spalding et al., 2007). Its barrier effects on population connectivity have been uncovered in several seep invertebrates, which show clear genetic break across the strait (Shen et al., 2016; Xiao et al., 2020; Xu et al., 2018, 2021). Our study provides incongruent results across species/OTUs. All the SCS species/OTUs are placed in four subclades distinct from each other, indicating multiple independent invasions of this genus to the SCS. The 655‐m species, *S. polita*, highly resembles *S. alucia* from the SW Atlantic. They form a pan‐oceanic lineage showing recent divergence between the SCS and SW Atlantic counterparts, and this lineage is likely to be restricted to the relatively shallow water depth (~ 600 m) (Shimabukuro et al., 2019). The detection of *S. balaenophila* lineage II (originally reported from the East Pacific) in the SCS suggests a potential trans‐Pacific distribution. The wide distributions of some whale‐fall invertebrates (e.g., *S. sirikos*, *S. balaenophila* lineage I [Shimabukuro et al., 2019] and *Osedax rubiplumus* [Zhou et al., 2020]) are attributed to their long‐distance dispersal capability, deduced from an elongated planktonic larval stage of these species (Rouse et al., 2009) or their relatives (Eckelbarger et al., 2001), and a relatively small distance between suitable substrates in the form of vertebrate carcasses, which are presumably in sufficient supply by widespread marine vertebrates (Rouse, Goffredi, et al., 2018). Thus, we speculate that the large populations of cetacea in the SCS, such as spinner dolphin (Lin et al., 2021; Liu et al., 2019; Xie et al., 2023), may act as stepping stones connecting cetacean falls on both sides of the LS. The lineage, formed by *S. nanhaiensis* and *S. feitiana*, is deeply divergent from all other congeners both genetically and morphologically. However, it is still too early to conclude that if the *S. nanhaiensis*/*S. feitiana* represents a SCS‐endemic lineage or not, which needs examinations on a wider spatial scale, especially in the West Pacific.

Species replacement along a depth‐related gradient have been reported at the community level in a wide variety of deep‐sea environments, including seamounts and trenches (Chivers et al., 2013; Kennedy et al., 2019; Shen et al., 2021; Victorero et al., 2018; Zhang et al., 2021), but has been rarely studied on closely related taxa. Combining specimen examinations and metabarcoding analyses reveals a higher *Sirsoe* diversity toward the deeper sites. Meanwhile, each species/OTU appears to be restricted to single site, except for *S. nanhaiensis* (which is present in both deep sites). Although the generality of such a pattern in the SCS need to be examined in more locations and more taxon groups, the diversity gradient and restriction of species to a limited depth range observed herein are consistent with previous studies in the East Pacific and Southwest Atlantic, where both *Osedax* and *Sirsoe* diversity peaked below 1000 m and very few of them span a broad depth range (Rouse, Goffredi, et al., 2018; Shimabukuro et al., 2019). Moreover, when combined with the reconstructed phylogeny, the depth zonation pattern of *Sirsoe* can be interpreted in a dimension in addition to species richness and species turnover. The sister relationships revealed between *S. feitiana* (3400 m) and *S. nanhaiensis* (1604 and 3400 m), and between OTU36 (3400 m) and OTU416 (1604 m), may exemplify either sympatric speciation or allopatric speciation induced by bathymetric isolation, while the placement of shallower *S. polita* on a deeply divergent lineage indicates its origin distinct from the deep counterparts. As thus, our results suggest closer evolutionary relationships between habitants of the two deep sites (1604 and 3402 m) than that of between the shallow (655 m) and deep sites (1604 and 3402 m).

This zonation pattern of *Sirsoe* may be partially explained by physical oceanography in the SCS. As the SCS is a semi‐enclosed marginal sea, water exchange between the SCS and surrounding seas is mainly driven by a three‐dimensional circulation, while the LS is the key connection between the SCS and the western Pacific Ocean. The upper layer inflow (<750 m) of LS was induced by wind stress and westward intrusion of the Kuroshio Current, the middle layer outflow (750–1500 m) and deep layer inflow (>1500 m) were influenced by the topographic effects and interior dynamical adjustment (Cai et al., 2020; Gan et al., 2022). The vertical inflow–outflow–inflow through the LS is one of the main driving forces inducing a cyclonic‐anticyclonic‐cyclonic circulation of the SCS, and consequently results in the formation of water masses governing distinct depth range and weak vertical mixing except for areas over the slope in the north and south, where topography–current interaction invokes intensified mixing (Cai et al., 2020). At a finer vertical scale, two water masses exist below 1000 m, with the boundary at about 2700 m (Fengqi et al., 2002). According to this physical scheme, the three deployed cow falls, from shallow to deep, are located in three distinct water masses, the Intermediate Water Mass, the Deep Water Mass and the Bottom Water Mass (Fengqi et al., 2002), respectively. Combined with the phylogenetic structure, we speculate that colonization of *Sirsoe* speices to the upper (<750 m) and deep layer (>1000 m) depth range might be induced via distinct water mass and the strong depth‐related gradient between the two layers may act as dispersal barrier preventing vertical admixture of them. And the water mass subdivision below the 1000 m may also contribute to the differentiation between the 1604‐ and 3402‐m cow falls.

Biotic factors may also contribute to the zonation pattern. One notable phenomenon observed during our investigations of the three cow falls was that the 655‐m site was visually dominated by another worm in the family Chrysopetalidae compared with the two deeper sites, where *Sirsoe* speices predominated the communities (Zhou, Y., Xie, W., Yin, K., & Zhang, D., unpublished data). Although in need of further evidence, we speculate that *Sirsoe* may either prefer deep‐sea environments, or be outcompeted by other more competent invertebrates in shallower water environments. Co‐occurrence of multiple *Sirosoe* species/OTUs at the two deep sites might be attributed to either niche partition in food, space or time between relatives, mechanism of which has got supports from studies on isotopic diet analyses and been used to explain the coexistence of relatives observed in hesinoids and dorvilleids (Alfaro‐Lucas et al., 2018; Thornhill et al., 2012). In addition, the temporal large amount of food (represented by mammal carcasses) can support high number of individuals feeding on them and may consequently allow coexistence of more species (Worm & Tittensor, 2018).

## AUTHOR CONTRIBUTIONS

**Yadong Zhou:** Conceptualization (equal); data curation (lead); formal analysis (lead); writing – original draft (lead); writing – review and editing (equal). **Yuru Han:** Data curation (equal); formal analysis (equal); methodology (equal); software (equal). **Wei Xie:** Funding acquisition (equal); investigation (equal); project administration (equal); resources (equal); supervision (equal); writing – review and editing (equal). **Mingting Li:** Data curation (equal); formal analysis (equal); methodology (equal); software (equal); visualization (equal); writing – original draft (equal). **Zhi Wang:** Formal analysis (equal); methodology (equal); writing – original draft (equal). **Dongsheng Zhang:** Conceptualization (equal); investigation (equal); project administration (equal); supervision (equal); writing – original draft (supporting); writing – review and editing (equal).

## FUNDING INFORMATION

This work was supported by the Innovation Group Project of Southern Marine Science and Engineering Guangdong Laboratory (Zhuhai), Grant Number: 3110220 and Southern Marine Science and Engineering Guangdong Laboratory (Zhuhai), Grant Number: SML2021SP309.

## CONFLICT OF INTEREST STATEMENT

The authors declare no conflict of interest.

## Data Availability

DNA sequences: GenBank accession numbers provided in Tables 2 and 3.
